# Captopril challenge test in the diagnosis of primary aldosteronism: consistency between 1- and 2- h sampling

**DOI:** 10.3389/fendo.2023.1183161

**Published:** 2023-06-12

**Authors:** Xinyu Liu, Chao Guo, Jin Bian, Sufang Hao, Ying Lou, Huimin Zhang, Xianliang Zhou, Jun Cai, Wenjun Ma

**Affiliations:** Hypertension Center, Fuwai Hospital, National Center for Cardiovascular Diseases, Chinese Academy of Medical Sciences and Peking Union Medical College, Beijing, China

**Keywords:** primary aldosteronism, captopril challenge test, hypertension, aldosterone concentration, confirmatory test

## Abstract

**Objective:**

To examine the consistency of plasma aldosterone concentration at 1 and 2 h in the captopril challenge test (CCT) and to explore the possibility of replacing 2-h aldosterone concentration with 1-h aldosterone concentration for diagnosis of primary aldosteronism (PA).

**Methods:**

This retrospective analysis included a total of 204 hypertensive patients suspected of having PA. Subjects received oral captopril challenge at 50 mg (25 mg if the systolic blood pressure was <120 mmHg), and plasma aldosterone concentration and direct renin concentration were measured at 1 and 2 h afterward (chemiluminescence immunoassay Liaison® DiaSorin, Italy). Sensitivity and specificity were used to reflect the diagnostic performance of 1-h aldosterone concentration using 2-h aldosterone concentration (11 ng/dl as the cutoff) as the reference. A receiver operating characteristic curve analysis was also conducted.

**Results:**

Among the 204 included patients [median age of 57.0 (48.0–61.0) years, 54.4% men], a diagnosis of PA was established in 94 patients. Aldosterone concentration in the patients with essential hypertension was 8.40 (interquartile range 7.05–11.00) ng/dl at 1 h and 7.65 (5.98–9.30) ng/dl at 2 h (*P* < 0.001). In patients with PA, aldosterone concentration was 16.80 (12.58–20.50) ng/dl at 1 h and 15.55 (12.60–20.85) ng/dl at 2 h (*P* > 0.999). At a cutoff of 11 ng/dl, the sensitivity and specificity of using 1-h aldosterone concentration to diagnose PA were 87.2% and 78.2%, respectively. A higher cutoff of 12.5 ng/ml increased specificity to 90.0% but decreased sensitivity to 75.5%. A lower cutoff of 9.3 ng/ml increased sensitivity to 97.9% but decreased specificity to 65.4%.

**Conclusions:**

When diagnosing PA with CCT, 1-h aldosterone concentration could not be used to replace 2-h aldosterone concentration.

## Introduction

Primary aldosteronism (PA) is one of the most common causes of secondary hypertension; it occurs in 5%–10% of hypertensive patients (1). PA involves spontaneous hypersecretion of aldosterone from the adrenal cortex, leading to increased plasma aldosterone concentration and renin suppression (2). Patients with PA are at higher risk of cardiovascular complications and renal damage than those with essential hypertension (EH), even after adjusting for age and blood pressure (3). PA can increase risk of morbidity and mortality even after initiation of medical treatment to control blood pressure and blocking the mineralocorticoid receptor (4). Thus, early diagnosis and intervention are crucial.

The diagnosis of PA typically consists of three steps: initial screening, confirmatory/exclusion testing, and subtyping (unilateral vs. bilateral disease) (5). Plasma aldosterone-to-renin ratio (ARR) is the most commonly used method for screening (6). Confirmatory tests include the fludrocortisone suppression test, saline infusion test, oral sodium loading test, and captopril challenge test (CCT) (5). Fludrocortisone suppression test is the most reliable but expensive and labor-intensive. The saline infusion test is simple but contradicted in patients with congestive heart failure and severe hypertension. The oral sodium loading test is least expensive but time-consuming and associated with heart failure and severe arrhythmia.

The CCT does not induce sharp fluctuations in blood pressure and therefore can be used in patients with severe hypertension and cardiac dysfunction; it is not significantly influenced by daily sodium intake (7). The CCT has been shown to be equally accurate as the fludrocortisone suppression test and saline infusion test (8–10).

Variables derived from CCT included aldosterone concentration, aldosterone concentration inhibition rate, and ARR. Among them, aldosterone concentration has the best diagnostic performance and thus recommended (10–12). However, there is lack of consensus on the timing of blood sampling after captopril challenge (5, 6, 13–15). For example, the Japan Endocrine Society recommends drawing blood at 1 or 1.5 h after captopril challenge (14) and the American Endocrine Society recommends blood sampling at 1 or 2 h after the challenge (5), whereas the Working Group on Endocrine Hypertension of the European Society of Hypertension and the Italian Society of Arterial Hypertension recommends sampling only at 2 h after captopril challenge (6, 13).

We conducted a retrospective analysis to examine the consistency between the data derived from 1 vs. 2 h after captopril challenge. In other words, the aim of the current study was to examine whether the CCT could be shortened to 1 h.

## Material and methods

### Study subjects

We retrospectively screened all hypertensive patients suspected of having PA and undergoing CCT at the Hypertension Center of the National Center for Cardiovascular Diseases/Fuwai Hospital (Beijing, China) during a period from 1 January 2019 to 31 July 2022. The study was approved by the Institutional Ethics Committee of Fuwai Hospital.

For inclusion in the final analysis, patients must have elevated ARR > 2.4 (ng/dl)/(μIU/ml) and underwent a standard CCT. Patients were excluded from the analysis if they were diagnosed with secondary hypertension other than PA, or currently taking steroids or oral contraceptives, or if they were preparing for pregnancy, already pregnant, or lactating.

Initial screening (determination of the ARR) followed the current guidelines (2, 5, 15) and was conducted at least 1 h after subjects had been in an upright position. Indication for the screening at our center included (1) blood pressure > 150/100 mmHg on each of three measurements obtained on different days; (2) hypertension (blood pressure >140/90mmHg) resistant to three conventional antihypertensive drugs (one must be a diuretic); (3) four or more antihypertensive drugs had to be combined to control blood pressure below 140/90 mmHg (4) hypertension with spontaneous or diuretic-induced hypokalemia; (5) hypertension with adrenal incidentaloma; (6) hypertension with a family history of early onset hypertension or cerebrovascular accident at a young age (<40 years old); and (7) all hypertensive first-degree relatives of patients with PA (2, 5, 15). Mineralocorticoid receptor antagonists and potassium-wasting diuretics were discontinued for at least 4 weeks, and other agents that could strongly affect ARR were withdrawn for at least 2 weeks prior to the screening. Furthermore, hypokalemia was corrected prior to the testing. Sodium intake was not restricted.

Patients with ARR >2.4 (ng/dl)/(μIU/ml) proceeded to the CCT. ARR > 2.4 (ng/dl)/(μIU/ml) was chosen to be the cutoff according to the 2016 guideline of the American Endocrine Society and the 2020 guideline of European Society of Hypertension (5, 6). The CCT was conducted at least 1 h after subjects had been in an upright position. Dosage of oral captopril challenge was 50 mg (25 mg if systolic pressure was <120 mmHg). Diagnosis of PA was made using plasma aldosterone concentration >11 ng/dl at 2 h after captopril challenge (6, 8). Subjects remained upright throughout the test.

### Biochemical measurements

Plasma aldosterone concentration and direct renin concentration were measured by the chemiluminescence immunoassay (Liaison® DiaSorin, Italy). Intra- and interassay coefficients of variability for plasma aldosterone concentration and direct renin concentration were both <5%.

### Management of hypertension

Severe uncontrolled hypertension was treated with a non-dihydropyridine calcium channel blocker, terazosin, doxazosin, and intravenous urapidil or nitroprusside if necessary.

### Statistical analysis

Continuous variables were analyzed for normal distribution using the Shapiro–Wilk test. Normally distributed variables were analyzed using Student’s *t*-test and expressed as mean ± standard deviation (SD). Variables not following normal distribution were analyzed using the Mann–Whitney U test, and expressed as median and interquartile range (IQR). Categorical variables were analyzed using the χ^2^ test and expressed as percentage.

Differences in aldosterone concentration, renin concentration, and aldosterone-to-renin ratio (ARR) among baseline and 1 and 2 h after the CCT were assessed using the Friedman test. Differences between 1 and 2 h in the CCT were analyzed using the Wilcoxon signed rank test with Bonferroni correction. The relationship between aldosterone concentration at 1 and 2 h in the CCT was assessed using Pearson correlation analysis.

Aldosterone concentration at 1 and 2 h in the CCT was compared using MedCalc 15.2 (MedCalc Software, Ostend, Belgium). Aldosterone concentration change, defined as the average difference between aldosterone concentration at 1 and 2 h (with 2-h aldosterone concentration as the reference), was assessed using Bland–Altman analysis (16). The accuracy of 1-h aldosterone concentration in diagnosing PA was assessed using sensitivity, specificity, Youden index, positive predictive value, negative predictive value, positive likelihood ratio, negative likelihood ratio, and area under the receiver operating characteristic curve (AUROC). When appropriate, 95% confidence intervals (CIs) were calculated.

Except indicated otherwise, statistical analyses were performed using SPSS 23.0 (IBM, Chicago, IL, USA). *P* < 0.05 (two-sided) was considered statistically significant.

## Results

### Clinicodemographic characteristics

The final analysis included 204 patients (54.4% men) with a median age of 57.0 (48.0–61.0) years. Based on aldosterone concentration at 2 h in the CCT, PA was established in 94 subjects (**Table 1**). In comparison to the essential hypertension (EH) group, the PA group had longer duration of hypertension, higher blood pressure (both systolic and diastolic), and lower serum potassium but higher urine potassium and higher serum creatinine.

**Table 1:** Demographic and clinical characteristics in patients with primary aldosteronism (PA) and essential hypertension (EH).

Characteristic	EH group (n = 110)	PA group (n = 94)	*P*
Age (years)	57.53 (48.00–61.00)	56.50 (49.02–61.25)	0.964
Male	60.0 (54.5)	51.0 (54.3)	0.967
HT duration (years)	7.50 (2.00–15.00)	11.00 (5.00–20.00)	**0.003**
SBP (mmHg)	136.94 ± 15.80	142.95 ± 17.36	**0.010**
DBP (mmHg)	81.16 ± 11.66	86.32 ± 10.30	**0.001**
FBG (mmol/L)	5.32 (4.75–5.82)	5.55 (4.90–6.14)	0.111
Serum K^+^ (mmol/L)	3.97 (3.77–4.17)	3.70 (3.35–4.09)	**< 0.001**
Serum Na^+^ (mmol/L)	143.27 (142.09–144.52)	143.35 (142.17–144.53)	0.488
Urine K^+^ (mmol/24 h)	35.71 (29.48–43.11)	40.49 (30.20–55.48)	**0.016**
Urine Na^+^ (mmol/24 h)	144.87 (104.13–185.75)	138.63 (95.09–203.83)	0.824
sCr (μmol/L)	72.69 (63.50–85.12)	79.66 (69.60–99.46)	**0.013**
ALT (U/L)	20.00 (16.00–30.50)	20.00 (15.00–27.00)	0.377

Data are n (%), mean ± standard deviation or median (25th–75th percentile), unless otherwise noted.

P values for significant differences between the two groups are marked in boldface.

ALT, alanine transaminase; DBP, diastolic blood pressure; FPG, fasting plasma glucose; HT, hypertension; SBP, systolic blood pressure; sCr, serum creatinine; serum K+, concentration of potassium in serum; serum Na^+^, concentration of sodium in serum; urine K^+^, concentration of potassium in urine; urine Na^+^, concentration of sodium in urine.

### Plasma aldosterone concentration, direct renin concentration, and aldosterone-to-renin ratio before and after captopril challenge

**Figure 1:**
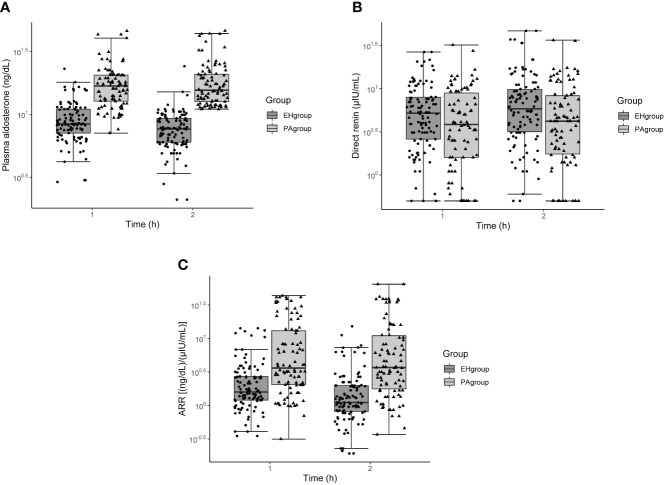
Box and whisker plots of plasma aldosterone concentration **(A)**, direct renin concentration **(B)**, and aldosterone-to-renin ratio **(C)** before and after the captopril challenge test in patients with primary aldosteronism or essential hypertension. The x-axis refers to time after captopril challenge.

In both the PA and EH groups, aldosterone concentration, renin concentration, and ARR differed significantly across the three time points examined: baseline and 1 and 2 h after captopril challenge (all *P* < 0.001). Within the EH group, but not the PA group, aldosterone concentration and ARR decreased while renin concentration increased from 1 to 2 h (all *P* < 0.05, **Table 2**). All three measures at 1 h overlapped between the PA and EH groups (**Figure 1**).

**Table 2:** Changes of plasma aldosterone concentration, direct renin concentration and aldosterone-to-renin ratio before and after the captopril challenge test in the PA and EH groups.

	Baseline	1 h after captopril challenge	2 h after captopril challenge	*P* (1 h vs. 2 h)
PA group				
aldosterone concentration (ng/dl)	20.00 (15.00–24.75)	16.80 (12.58–20.50)	15.55 (12.60–20.85)	>0.999
renin concentration (uIU/ml)	2.10 (0.78–4.00)	3.85 (1.60–9.30)	4.20 (1.70–8.38)	0.465
ARR [(ng/dl)/(μIU/ml)]	7.96 (4.81–28.68)	3.62 (2.03–13.22)	3.67 (1.71–11.23)	>0.999
EH group				
aldosterone concentration (ng/dl)	12.15 (9.60–16.45)	8.40 (7.05–11.00)	7.65 (5.98–9.30)	**<0.001**
renin concentration (μIU/ml)	2.85 (1.40–4.12)	5.20 (2.58–8.00)	5.85 (3.10–9.90)	**0.015**
ARR [(ng/dl)/(μIU/ml)]	4.03 (3.14–6.76)	1.59 (1.20–2.71)	1.11 (0.81–1.98)	**<0.001**

Data are expressed as median (25th−75th percentile).

P values for significant differences between the two groups are marked in boldface.

ARR, the aldosterone-to-renin ratio; CCT, captopril challenge test; EH, essential hypertension; PA, primary aldosteronism.

### Consistency between 1- and 2- h data

**Figure 2:**
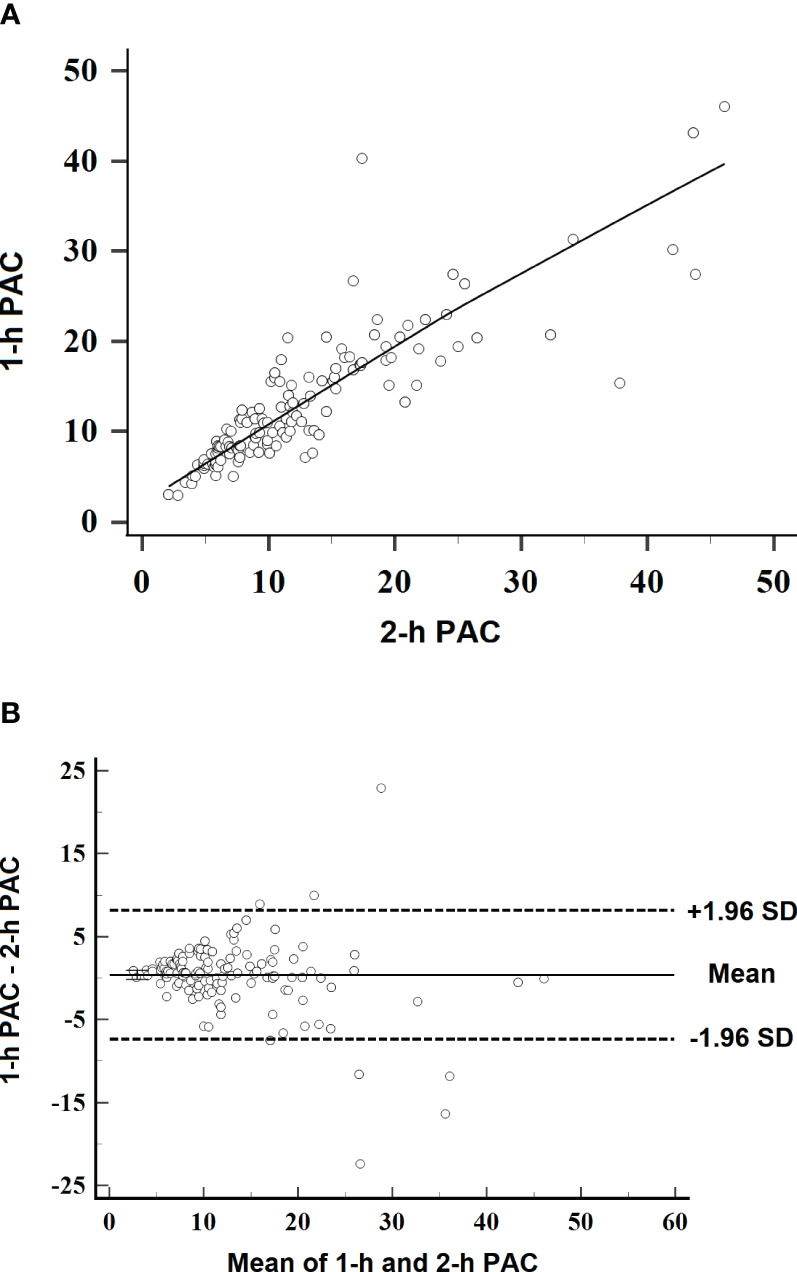
Scatterplots of linearity and variation in plasma aldosterone concentration at 1 h after captopril challenge. **(A)** Linearity between aldosterone concentrations at 1 and 2 h after captopril challenge. The line indicates the best-fit regression (r = 0.891, *P* < 0.001). **(B)** Bland–Altman plot of 1-h aldosterone concentration relative to 2-h aldosterone concentration as reference. SD, standard deviation.

Plasma aldosterone concentration at 1 h correlated with aldosterone concentration at 2 h (r = 0.891, *P* < 0.001; **Figure 2A**). In Bland–Altman analysis, aldosterone concentration change between the two time points was 0.398 ng/dl (95%CI, −7.35– 8.15 ng/dl) (**Figure 2B**).

In ROC analysis, AUROC representing the diagnostic accuracy of 1-h aldosterone concentration was 0.905 (95% CI 0.856–0.942). At a cutoff of 11 ng/dl (as in the standard 2-h CCT method), the sensitivity and specificity of 1-h aldosterone concentration were 87.2% and 78.2%, respectively (**Table 3**). A higher cutoff of 12.5 ng/ml increased specificity to 90.0% but decreased sensitivity to 75.5%. A lower cutoff of 9.3 ng/ml increased sensitivity to 97.9% but decreased specificity to 65.4%.

**Table 3:** Diagnostic accuracy of plasma aldosterone concentration assayed at 1 h after captopril challenge, based on different cutoffs.

Cutoff (ng/dl)	TP (N)	FP (N)	FN (N)	TN (N)	Youden index	Sensitivity (95% CI)	Specificity (95% CI)	PPV (%)	NPV (%)	PLR	NLR
> 9.3	92	38	2	72	0.633	97.9 (92.5–99.7)	65.4 (55.8–74.3)	70.77	97.30	2.83	0.033
> 10	85	32	9	78	0.613	90.4 (82.6–95.5)	70.9 (61.5–79.2)	72.65	89.66	3.11	0.14
> 11	82	24	12	86	0.654	87.2 (78.8–93.2)	78.2 (69.3–85.5)	77.36	87.76	4.00	0.16
> 12.1	73	15	21	95	0.640	77.7 (67.9–85.6)	86.4 (78.5–92.2)	82.95	81.90	5.70	0.26
> 12.5	71	11	23	99	0.655	75.5 (65.6–83.8)	90.0 (82.8–94.9)	86.59	81.15	7.55	0.27

FN, false negative; FP, false positive; NLR, negative likelihood ratio; NPV, negative predictive value; PLR, positive likelihood ratio; PPV, positive predictive value; TN, true negative; TP, true positive.

## Discussion

Results from the current study showed similar aldosterone concentration at 1 and 2 h after captopril challenge in PA patients but higher aldosterone concentration at 1 h than 2 h in EH patients. W e failed to find a 1-h aldosterone concentration cutoff value that offered both satisfactory sensitivity and specificity. Based on these results, we believe that 1-h aldosterone concentration could not be used to replace 2-h aldosterone concentration.

The CCT to diagnose PA is not standardized, especially in terms of sampling times after captopril administration. A recent prospective study conducted in South Korea found that at the cutoff value of 13 ng/dl, 1-h aldosterone concentration led to sensitivity and specificity of 98.0% and 64.3%, while 1.5-h aldosterone concentration led to sensitivity and specificity of 98.0% and 78.6% in diagnosing PA (17).

A previous study from our team compared the variation of the aldosterone concentration, renin concentration, and ARR at 1, 1.5, and 2 h after captopril challenge in 30 EH patients and 25 PA patients, and we found that there were no significant differences in aldosterone concentration, renin concentration, or ARR across 1, 1.5, and 2 h in either PA or EH patients (18). In that study, 1- and 1.5-h aldosterone concentration produced 90.0% and 95.0% sensitivity, 92.0% and 96.0% specificity, and 0.945 and 0.970 AUROC at an aldosterone concentration cutoff of 9.95 ng/dl (18). Therefore we reviewed more patients in the present study. The results of the current study, however, showed that 1-h aldosterone concentration was significantly higher than 2-h aldosterone concentration in EH patients. This difference from our previous pilot study likely reflects the larger sample in the present work.

In the Bland– Altman plot, the 95% CI of aldosterone concentration change was −7.35– 8.15 ng/dl. Such a wide range is clearly not acceptable clinically for replacing 2-h aldosterone concentration for the diagnosis of PA.

Despite the promising AUROC of 1-h aldosterone concentration at 0.905, we failed to find an aldosterone concentration cutoff value that offered both satisfactory sensitivity and specificity. When using 11 ng/dl as the cutoff, the sensitivity and specificity were 87.2% and 78.2%. A higher cutoff of 12.5 ng/ml increased specificity to 90.0%, but decreased sensitivity to 75.5%. A lower cutoff of 9.3 ng/ml increased sensitivity to 97.9% but decreased specificity to 65.4%. In light of these results, we conclude that 1-h aldosterone concentration could not be used to replace the 2-h data in the diagnosis of PA.

Captopril reduces serum aldosterone and increases serum renin in patients without autonomous aldosterone secretion but not in patients with PA (19). The effects of captopril are maximal at 60–90 min after oral administration, with 180-min half-life (20). This may explain why we observed lower aldosterone concentration at 2 h than at 1 h in EH patients. In PA patients, serum aldosterone was not inhibited; accordingly, aldosterone concentration was similar between 1 and 2 h in PA patients.

The current study has several limitations. First, the study was conducted at a single center and in a relatively small sample. Second, we did not conduct a saline infusion test or a fludrocortisone suppression test to benchmark the diagnostic performance of 1- or 2-h aldosterone concentration. Third, adrenal venous blood was not sampled to determine PA subtypes. Fourth, this is a retrospective study with unknown bias. Prospective studies of large sample size and a more solid reference test are needed.

In summary, the results from the current study showed that 1-h aldosterone concentration could not be used to replace 2-h aldosterone concentration in CCT.

## Data availability statement

The raw data supporting the conclusions of this article will be made available by the authors, without undue reservation.

## Ethics statement

The studies involving human participants were reviewed and approved by Institutional Ethics Committee of Fuwai Hospital. The patients/participants provided their written informed consent to participate in this study.

## Author contributions

WM contributed to conception and design of the study. JB and SH organized the database. YL and CG performed the statistical analysis. XL wrote the first draft of the manuscript. XZ, HZ, and JC wrote sections of the manuscript. All authors contributed to manuscript revision, read, and approved the submitted version.
